# Successful surgical treatment of Cronkhite-Canada Syndrome with bilateral flail chest: a case report

**DOI:** 10.1186/s12893-020-00766-z

**Published:** 2020-05-13

**Authors:** Guang-chao Lv, Zhi-hong Li, Zong-sheng Duan, Chun-bo Niu, Ming-he Li, Kai-zhong Wang, Jin-dong Jiang

**Affiliations:** 1 Department of Thoracic Surgery, First Hospital of Jilin University, No. 71, Xinmin Street, Changchun, Jilin People’s Republic of China; 2 Department of Anesthesiology, First Hospital of Jilin University, No. 71, Xinmin Street, Changchun, Jilin People’s Republic of China; 3 Department of Pathology, China-Japan Union Hospital of Jilin University, No. 829 Xinmin Street, Changchun, Jilin People’s Republic of China; 4 Department of Oral and Maxillofacial Surgery, School of Stomatology Hospital of Jilin University, Changchun, 130021 China

**Keywords:** Cronkhite–Canada syndrome (CCS), Flail chest, Multiple rib fractures

## Abstract

**Background:**

Development of multiple rib fractures leading to bilateral flail chest in Cronkhite–Canada Syndrome (CCS) has not been reported.

**Case presentation:**

A 59-year-old man presented with complaints of fatigue, chest pain, respiratory distress and orthopnea requiring ventilatory support to maintain oxygenation. CCS with bilateral anterior and posterior flail chest due to multiple rib fractures (2nd-10th on the right side and 2nd-11th on the left side). He underwent open reduction and anterior and posterior internal fixation using a titanium alloy fixator and a nickel-titanium memory alloy embracing fixator for chest wall reconstruction. He recovered gradually from the ventilator and showed improvement in his symptoms. He gained about 20 kg of weight in the follow up period (6 months after discharge from the hospital).

**Conclusion:**

CCS is a rare, complex disease that increases the risk of developing multiple rib fractures, which can be successfully treated with open reduction and internal fixation.

## Background

Cronkhite–Canada Syndrome (CCS) is a rare, non-familial disease presenting with diarrhea, weight loss, alopecia, multiple gastrointestinal polyposis, onychodystrophy and hyperpigmentation [1]. Since the first description in 1955, over 500 cases have been reported worldwide [2]. CCS predominantly affects males between 50 and 60 years of age [3]. It is associated with poor long-term survival [4]. Medical treatment includes corticosteroids, nutritional supplementation, antibiotics, non-steroidal anti-inflammatory drugs (NSAIDs), proton pump inhibitors (PPIs) and histamine H2 receptor antagonists [5, 6]. Surgery is usually reserved for treatment of complications. To the best of our knowledge, development of bilateral anterior and posterior flail chest in CCS and its surgical treatment has not been reported in the literature. We, herein, report a case of CCS with bilateral flail chest that was treated successfully with open reduction and internal fixation.

## Case presentation

A 59-year-old man presented with complaints of fatigue, respiratory distress, orthopnea and failure to thrive. He had a history of multiple rib fractures (2nd-10th on the right side and 2nd-11th on the left side) due to excessive body massage 1 year previously, leading to bilateral flail chest. He also had diarrhea (2–3 times per day), weight loss of approximately 15 kg over 2 years and onychodystrophy involving the finger and toe nails. He had undergone left hemicolectomy 1 year prior for multiple gastrointestinal polyps at another hospital and was diagnosed with CCS based on histopathological examination. He had no family history of polyposis. Since that time, he has been treated with nutritional supplementation, antibiotics (levofloxacin and cefepime) and corticosteroids (hydroprednisone) for 6 months.

On clinical examination, the patient was malnourished, emaciated and had loss of finger and toe nails. He could not lay down to sleep. He had a partial arterial oxygen pressure of 58 mmHg and an oxygen saturation (SpO_2_) of 88% with nasal oxygen. He had paradoxical chest movements on both sides due to flail chest and pseudarthrosis.

**Fig. 1:**
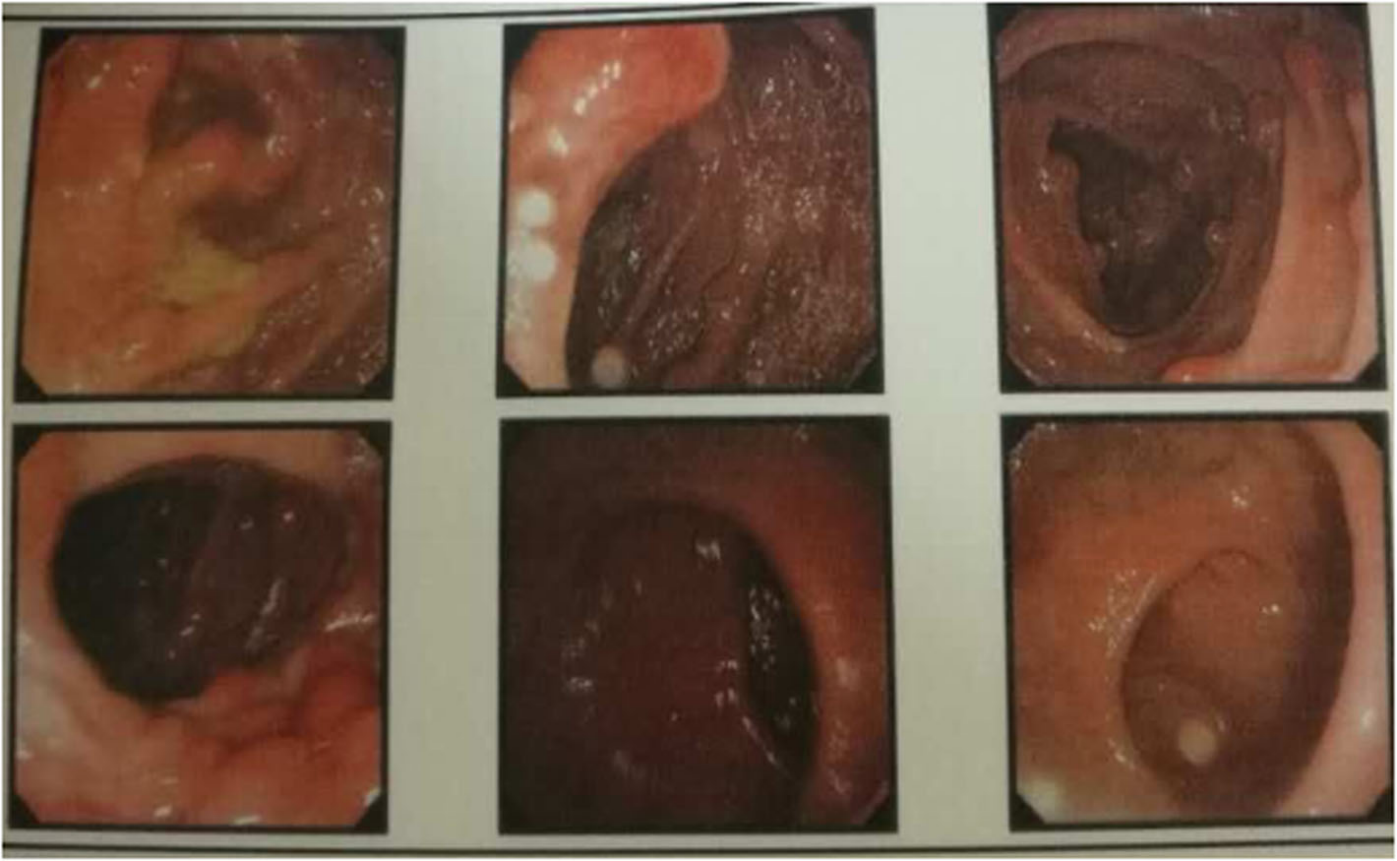
Colonoscopy found more than 100 polyps diffusely distributed throughout the colon starting from the anus up to about 17 cm from the cecum. The size of the polyps varied from 0.8–1.5 cm with inflamed mucosa and the presence of a few erosions

**Fig. 2:**
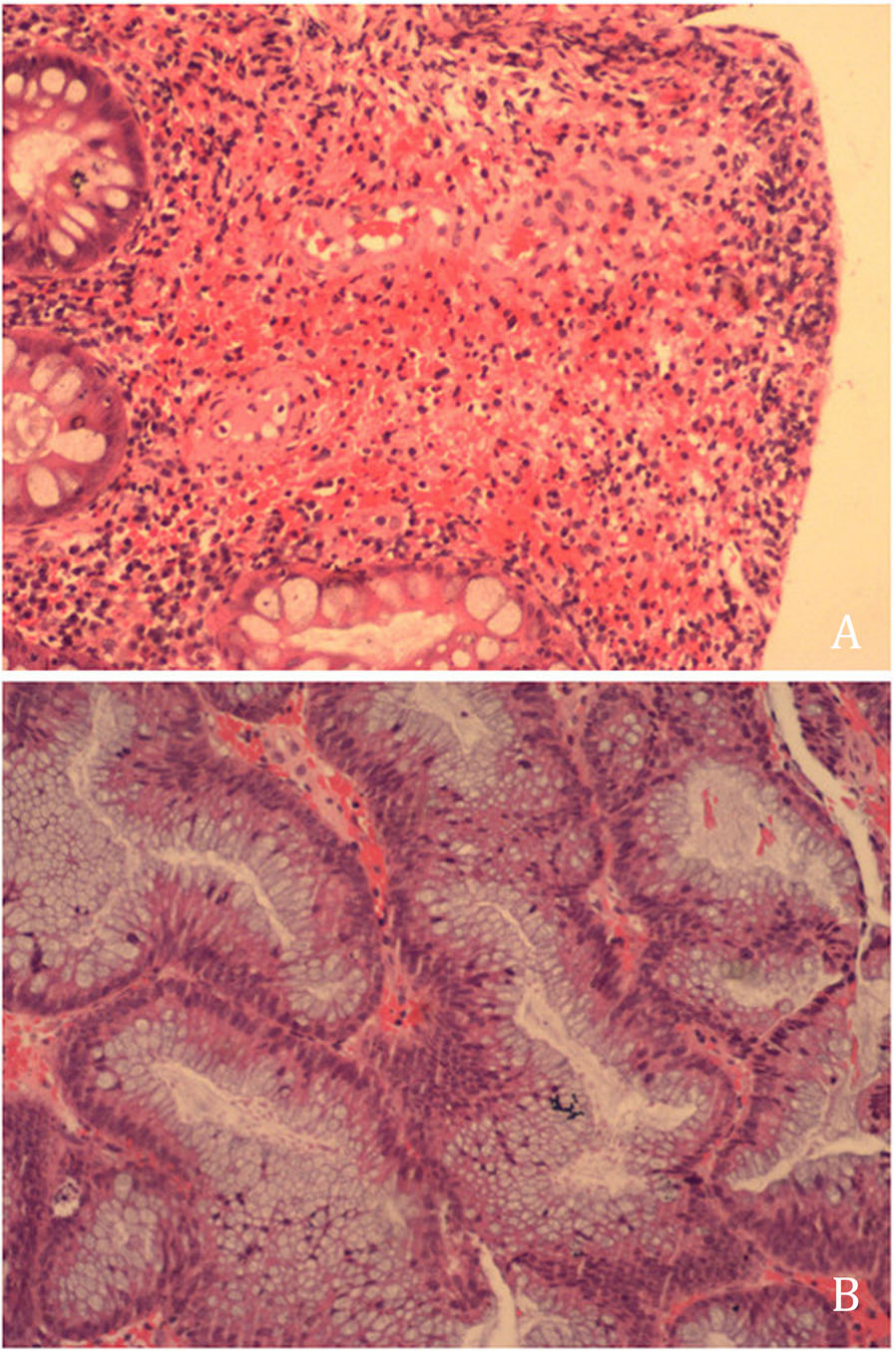
**a**. Histopathological examination of the polyps revealed adenomatous changes, mild atypical hyperplasia and the presence of inflammatory granulomas as seen on hematoxylin and eosin (HE) staining (magnification, 100×) (**a**). **b**. On immunohistochemical analysis, the polyps were IgG (+) and IgG4 (−)

**Fig. 3:**
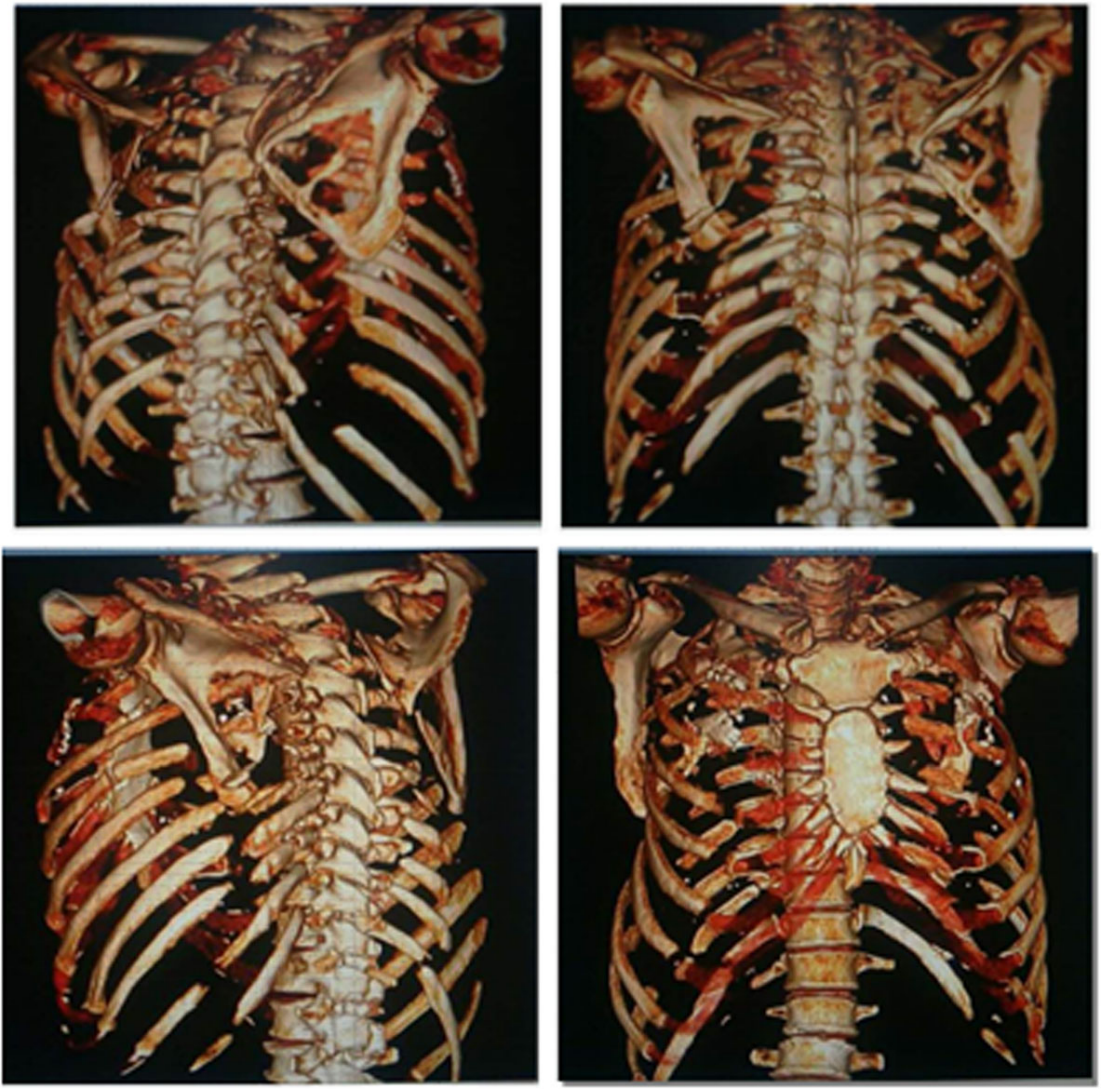
Chest CT (3D reconstruction): bilateral anterior and posterior multiple rib fractures (2nd-10th on the right side and 2nd-11th on the left side)

On colonoscopy, we found more than 100 colonic polyps diffusely distributed, starting at 17 cm from the cecum up to the anus with inflamed mucosa and few erosions in the size range of 0.8–1.5 cm (Fig. 1). Histopathological evaluation of the colonic biopsy revealed multiple adenomatous polyps, mild atypical hyperplasia and inflammatory granuloma (Fig. 2a and Fig. 2b). On immunohistochemical analysis, IgG staining was present but staining for IgG4 was negative. Computed tomography (CT) of the chest showed multiple fractures of the 2nd-10th ribs on the right side and the 2nd-11th ribs on the left side (Fig. 3). Laboratory investigations showed a serum albumin concentration of 31 g/L (normal range, 40–60 g/L), serum calcium concentration of 2.04 mmol/L (normal range 2.25–2.75 mmol/L), serum phosphorous concentration of 14 mmol/L, serum ferritin concentration of 225 ng/ml, highly sensitive C-reactive protein (hsCRP) concentration of 5.48 mg/L, and an erythrocyte sedimentation rate (ESR) of 7 mn/h. The T-spot/TB test yielded 176 + 28^FC^ /10S6MC. Evaluation of tumor markers showed a carcinoembryonic antigen (CEA) level of 5.17 ng/ml with the absence of alpha-fetoprotein (AFP), CA19–9, and CA242. The kidney androgen-regulated protein (KAP) level was 7.55 mg/dL, and the results of the human lipoarabinomannan assay (for tuberculosis) were normal. The serum immunoelectrophoresis test was negative, the immunoglobulin 4 (IgG4) concentration was 5940 ng/mL, Ig alexin was normal. Analysis of antinuclear antibodies (ANAs) revealed the patient was PCNA(+) and anti-ENA(−). Analysis of bone metabolism showed a β-CTX (β-Crosslaps for bone resorption) concentration of 0.621 ng/mL, a T-25-OH-vitaminD concentration of 7.4 ng/mL, and a parathyroid hormone (PTH) concentration of 94.2 pg/mL. The patient had severe osteoporosis and osteomalacia.

**Fig. 4:**
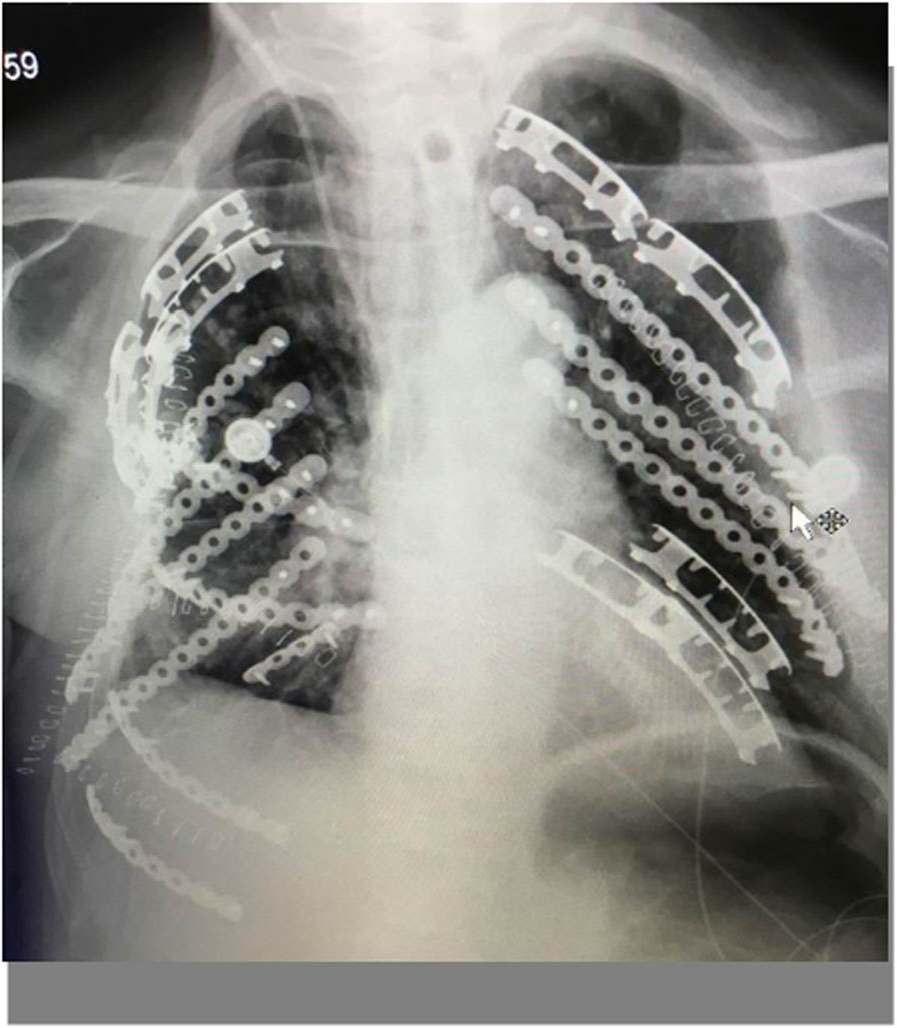
Digital radiograph showing the titanium alloy fixators and nickel-titanium memory alloy embracing fixators used to treat the multiple rib fractures

**Fig. 5:**
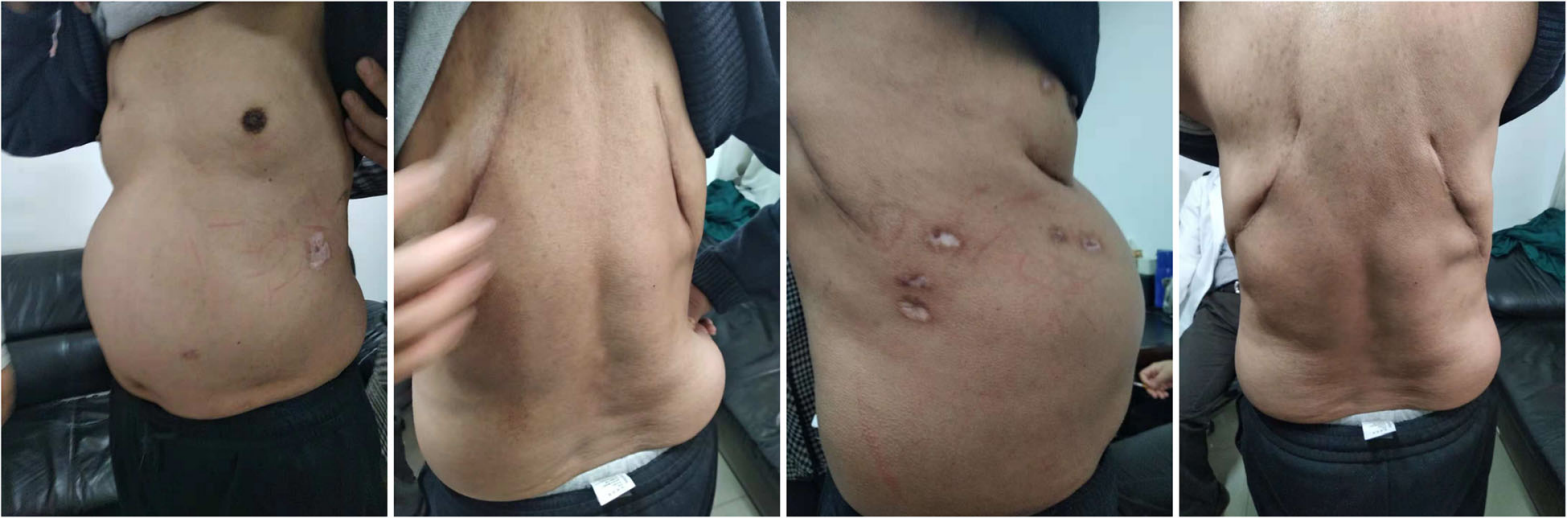
One year later, the patient’s body weight had increased by 20 Kg

We continued his nutritional supplementation and antibiotics but stopped his steroid treatment due to the rib fractures. He underwent open reduction and internal fixation twice (anterior and posterior separately) using a titanium alloy fixator and a nickel-titanium memory alloy embracing fixator for chest wall reconstruction (Fig. 4). The surgeries were done in two sessions because the first operation took a long time and the patient could not tolerate the prolonged anesthesia. The fixations were done using two bilateral curved incisions one on either side. We mainly retracted the muscles during the fixation and muscle cutting was performed only at few places. Postoperatively, he developed respiratory muscle weakness and respiratory failure due to which he required prolonged ventilator support. Tracheostomy and bronchial lavage were performed to provide ventilator-assisted breathing after operation. He recovered gradually and remained on the invasive ventilator for 3 months (2 months in hospital and 1 month at home). His hospital stay was 2 months. Subsequently, he received nutritional support at home and household noninvasive ventilator support for 6 months. At last follow-up (6 months after discharge), the patient’s body weight had increased by 20 kg and the patient showed improvement in his symptoms (Fig. 5).

## Discussion and conclusions

CCS, is characterized by gastrointestinal polyposis and skin changes [7]. Most of the cases have been reported from Japan [8, 9]. The exact etiology of CCS is not known. Some possible risk factors include genetic abnormalities [10], mental stress [11], and immune dysregulation [7, 12]. The male: female ratio is 3:2. The most common age at presentation is 6th decade of life [3]. The overall prognosis of CCS is poor with a 5-year mortality rate of 55%. The majority of deaths are associated with malnutrition, repetitive infections, heart failure, gastrointestinal bleeding and life-threatening malignant complications [13, 14].

The most common initial symptoms are diarrhea, anemia, edema, weight loss, and changes in the skin and nails. Ectodermal changes are frequently seen several weeks or months after the appearance of gastrointestinal symptoms. Endoscopy is the investigative modality of choice. Typically, sessile polyps of various sizes can be seen distributed throughout the gastrointestinal tract [7]. On histopathological examination, these polyps may be adenomatous, juvenile or inflammatory-type [9]. Classically, IgG4-positive inflammatory cell infiltration is present with edema in the lamina propria [13]. However, in our case, staining for IgG was positive, but that for IgG4 was negative. The diagnosis of CCA is based on the presence of multiple gastrointestinal inflammatory polyps, alopecia, onychodystrophy and hyperpigmentation [15]. The medical therapy includes corticosteroids, nutritional supplementation, antibiotics, NSAID, and PPIs [16]. Steroids are considered the main stay of medical treatment, although the recommended doses and durations vary widely in the literature, with no current gold standard [9, 14]. However, CCS patients who receive long-term treatment with corticosteroids usually develop osteoporosis and have an increased risk of rib fractures [17]. In addition, hypocalcemia and malnutrition can predispose to the development of rib fractures. Hence, patients with CCS on corticosteroid therapy should be regularly screened for potential complications such as rib fracture [17].

The flail chest is said to be present if there are fracture of ≥3 consecutive ribs at two places, with or without a sternal component, associated with chest wall paradoxical movements during respiration [18]. Patients with flail chest may develop pneumonia, post-traumatic deformity, chronic pain and may require assisted breathing with ventilator. The presence of underlying pulmonary parenchymal disease can further worsen the patient’s prognosis. Compared to lateral flail segments, anterior flail segments have a higher morbidity [19]. Treatment of flail chest includes oral, intravenous and/or epidural analgesia, oxygen therapy by mask or nasal prongs and if required mechanical ventilation [18, 19].

Although in most cases of thoracic trauma surgical reconstruction is not necessary, these patients often require prolonged mechanical ventilation [18, 19]. Osteosynthesis with plates stabilizes the chest wall. This helps in weaning off the patients of flail chest from mechanical ventilation accelerates the recovery. However, the precise role of surgical fixation in flail chest and the appropriate timing of surgery continues to be uncertain. Current indications for surgical fixation include: (1) thoracotomy for complicated by multiple injuries (especially other thoracic injuries), (2) failure to wean from mechanical ventilation, (3) severe chest wall instability, (4) persistent chronic pain, and (5) progressive respiratory dysfunction [20]. Previous reports suggest that surgical rib stabilization significantly reduces the duration of mechanical ventilation, hospital stay, incidence of pneumonia and tracheostomy compared to conservative treatment [21]. Furthermore, several studies have reported that surgical reconstruction can improve long-term pulmonary function and decrease pain compared to non-operative management [22].

Numerous surgical methods for rib fractures have been described in the literature [22, 23]. In our case, we used titanium alloy fixators and a nickel-titanium memory alloy embracing fixators. Osteosynthesis with plates has improved to be the most extensive security option for the surgical treatment of rib fractures [23]. The clinical implication of the flail segment can vary based on the size and the anatomical location. Precise preoperative planning is usually required based on three-dimensional reconstructed CT of the thorax, followed by precise planning of the surgical access routes [24]. However, the operative treatment of different patterns of chest wall injuries involving multiple rib fractures may be challenging.

Surgery is usually reserved for the treatment of complications. In the present case, the patient developed bilateral anterior and posterior flail chest due to multiple rib fractures (2nd-10th on the right side and 2nd-11th on the left side) and pseudarthrosis. Initially, the patient was managed conservatively by ventilator-assisted breathing, nutritional supplementation, antibiotics and corticosteroids for 6 months. However, due to persistent severe symptoms, the patient underwent open reduction and internal fixation of the rib fractures as well as reconstruction of the chest wall.

We report a rare case of CCS in which the patient developed bilateral flail chest (anterior and posterior) due to multiple rib fractures that was successfully treated by open reduction and internal fixation to reconstruct the chest wall after failed medical treatment. To the best of our knowledge, such a case has not been reported in the English literature. In this case, the exact cause of multiple rib fractures was difficult to pin-point, due to the presence of multiple factors such as CCS, steroid therapy and a history of trauma. Future studies are required to standardize the management of such complications in patients with CCS.

## Data Availability

The datasets generated and analyzed during the present study are available from the corresponding author on reasonable request.

## Abbreviations

CCS: Cronkhite–Canada Syndrome
NSAID: Nonsteroidal anti-inflammatory drugs
PPIs: Proton pump inhibitors
SpO_2_: Oxygen saturation
CT: Computed tomography
hsCRP: Highly sensitive C-reactive protein
ESR: Erythrocyte sedimentation rate
CEA: Carcinoembryonic antigen
AFP: Absence of alpha-fetoprotein
KAP: Kidney androgen-regulated protein
IgG4: Immunoglobulin 4
ANAs: Antinuclear antibodies
PCNA: Proliferating Cell Nuclear Antigen
anti-ENA: Anti-Extractable nuclear antigen
β-CTX: β-Crosslaps for bone resorption
PTH: Parathyroid hormone
